# Does anti-Mullerian hormone predict the outcome of further pregnancies in idiopathic recurrent miscarriage? A retrospective cohort study

**DOI:** 10.1007/s00404-018-4946-7

**Published:** 2018-10-24

**Authors:** Sophie Pils, Natalia Stepien, Christine Kurz, Kazem Nouri, Stephanie Springer, Marlene Hager, Regina Promberger, Johannes Ott

**Affiliations:** 1 Clinical Division of Gynecologic Endocrinology and Reproductive Medicine, Department of Obstetrics and Gynecology, Medical University of Vienna, Waehringer Guertel 18-20, 1090 Vienna, Austria; 2 Department of Obstetrics and Gynecology, Saint John of God Hospital Eisenstadt, Burgenland, Austria

**Keywords:** AMH, Idiopathic recurrent miscarriage, Maternal age

## Abstract

**Purpose:**

To evaluate whether anti-Mullerian hormone, basal follicle-stimulating hormone, luteinizing hormone, estradiol, and female age would predict future outcomes in women with idiopathic recurrent miscarriage.

**Methods:**

One hundred and sixteen women with idiopathic recurrent miscarriage were retrospectively included. Luteal support with or without a combined treatment regimen for idiopathic recurrent miscarriage was applied in a tertiary-care center in Vienna. Occurrence and outcome of further pregnancies were analyzed.

**Results:**

Within a median follow-up duration of 42.3 months, 94 women (81.0%) achieved one or more pregnancies. Further miscarriages occurred in 47 patients in whom only a higher number of previous miscarriages was predictive (OR 3.568, 95% CI 1.457–8.738; *p *= 0.005). Fifty-seven women had a live birth > 23 + 0 gestational weeks. In a multivariate analysis, age (OR 0.920, 95% CI 0.859–0.986; *p *= 0.019) and the number of previous miscarriages (OR 0.403, 95% CI 0.193–0.841; *p *= 0.016), but not AMH (OR 1.191, 95% CI 0.972–1.461; *p *= 0.091) were significantly predictive.

**Conclusion:**

AMH seems of either no or only minor relevance for the prediction of further miscarriages and live birth in women with idiopathic recurrent miscarriage.

## Introduction

Recent studies have demonstrated an association between anti-Mullerian hormone (AMH) levels and recurrent miscarriage (RM) [1], especially idiopathic recurrent miscarriage (IRM) [2], and the type of early pregnancy loss in IRM [3]. Although these findings suggest a possible future role of AMH in the diagnostic evaluation of RM/IRM, the main question of whether AMH testing would really predict further outcomes in these patients remains open.

Since a high rate of about 5–75% of miscarriages is associated with embryonic chromosomal abnormalities also caused by decreased oocyte quality [4, 5], and various studies have demonstrated increased rates of chromosomal abnormalities in embryos derived from couples with RM [6–10], it would seem reasonable that serum parameters for reproductive age could predict further outcomes of women with IRM, first and foremost, live births and the reoccurrence of miscarriage. Thus, to evaluate whether these were associated with serum levels of AMH, basal follicle-stimulating hormone (FSH), basal luteinizing hormone (LH), basal estradiol, and female age was the aim of the present study.

## Materials and methods

### Patient population and study design

Between January 2008 and July 2016, 134 women with IRM had undergone a complete diagnostic evaluation at our department and were included in this retrospective study. As reported previously [2, 3], RM was diagnosed in case of a documented history of at least three spontaneous, consecutive miscarriages before 20 weeks’ gestation, with the same partner. The standard diagnostic evaluation included: diagnostic hysteroscopy for exclusion of intrauterine synechia and uterine malformations; thrombophilia screening, including protein S antigen, protein C activity, APC-resistance, and antithrombin III activity; paternal and maternal karyotype; cervical cultures for chlamydia, ureaplasma, and mycoplasma; a comprehensive hormonal status panel that included thyroid-stimulating hormone (TSH) (ELECSYS^®^ TSH, Roche Diagnostics GmbH, Mannheim, Germany), prolactin (ELECSYS^®^ Prolactin II, Roche Diagnostics GmbH, Mannheim, Germany), testosterone (ELECSYS^®^ Testosterone II, Roche Diagnostics GmbH, Mannheim, Germany), androstenedione (IMMULITE^®^ 2000 Androstenedione, Siemens Healthcare Diagnostics Products Ltd., Llanberis, UK), dehydroepiandrosterone-sulfate (ELECSYS^®^ DHEA-S, Roche Diagnostics GmbH, Mannheim, Germany), and 17-hydroxy-progesterone (17-OH-Progesterone ELISA in Serum, IBL International Gmbh, Hamburg, Germany); evaluation of diabetes mellitus with HbA1c (D-100^tm^ HbA1c, BIO-RAD, Marnes-la-Coquette, France) assessment; evaluation of antiphospholipid syndrome with IgM and IgG anti-cardiolipin antibody (ORG 515 Anti-Cardiolipin IgG/IgM, ORGENTEC Diagnostika GmbH, Mainz, Germany) assessment (normal ranges < 10 IU/mL and < 7 IU/mL, respectively); and IgM and IgG anti-beta-2-glycoprotein I antibody assessment (ORG 521 Anti-beta-2-Gycoprotein I IgG/IgM ORG, ORGENTEC Diagnostika GmbH, Mainz, Germany) (normal ranges for both parameters < 5 IU/mL) [3, 11]. If no abnormalities were found using the above-mentioned tools, including polycystic ovary syndrome defined by the revised Rotterdam criteria [12], adrenogenital syndrome, hyperprolactinemia, and TSH levels > 2.5 IU/mL [2, 3], IRM was diagnosed. Patients who did not want to get pregnant anymore (*n* = 7), patients who were lost to follow-up (*n* = 10), and those with a subsequent termination of a further pregnancy due to trisomy 21 (*n* = 1) were excluded from the study. This resulted in a final population of 116 women. The routine treatment for IRM was called the “combined treatment regimen” and was recommended to all patients. It was an oral combination treatment consisting of prednisone (20 mg/day) and dydrogesterone (20 mg/day) for the first 12 weeks of gestation, aspirin (100 mg/day) for 38 weeks of gestation, and folate acid (5 mg) every second day throughout the pregnancies [11]. However, it was up to the affected women to follow these recommendations. Thus, our study population also included women who chose not to apply the combined treatment regimen, but who chose luteal support only with either oral dydrogesterone 10 mg twice a day or oral/vaginal progesterone 100 mg two–three times a day.

The study was approved by the Institutional Review Board (IRB)of the Medical University of Vienna (IRB number 2088/2016). Data in this retrospective study were anonymized; thus, there was no need for informed consent according to the regulations of the IRB. There was no funding.

### Parameters analyzed

For this study, the following parameters were retrieved by retrospective chart review. (1) The major outcome parameter was whether women managed to become pregnant again, and, in case of further pregnancies, the outcome defined as either a miscarriage or a live birth after 23 + 0 week of gestation. For this parameter, multiple selections were possible if the patient had experienced more than one further pregnancy. In case of a pregnancy, patients underwent regular follow-up examinations at our department until a heartbeat could be visualized on ultrasound. (2) Basal serum levels of FSH, LH, AMH, and estradiol. All of these blood samples had been obtained from a peripheral vein on menstrual cycle days 3–5 at the time of diagnostic evaluation of RM. All examined serum parameters had been determined in the ISO-certified central laboratory of the Vienna General Hospital, Austria. Following assays were used for estradiol: ELECSYS^®^ Estradiol III, Roche Diagnostics GmbH, Mannheim, Germany; LH: ELECSYS^®^ LH, Roche Diagnostics GmbH, Mannheim, Germany); FSH: ELECSYS^®^ FSH, Roche Diagnostics GmbH, Mannheim, Germany; and AMH: DSL Active MIS/AMH assay; Beckman Coulter Inc., Brea, USA. The AMH cut-off for poor ovarian reserve was defined as ≤ 1 ng/mL, as recommended previously [13]. (3) Patient age and body mass index (BMI) at the time of diagnostic evaluation. (4) The number of previously experienced miscarriages and whether women had experienced primary versus secondary RM (no versus at least one prior pregnancy exceeding a gestational age of 20 weeks before the series of pregnancy losses, respectively). (5) Ongoing treatment (combined treatment regimen vs. luteal support only. Data on intravenous immunoglobulin immunotherapy were not analyzed, since a recent meta-analysis showed insufficient evidence on the effect of this treatment [14]. Parameters (2)–(5) were included as predictive factors in the multivariate models.

### Statistical analysis

Data are presented as numbers and frequencies for categorical and as median and interquartile range (IQR) for continuous variables. Statistical analyses were performed with the SPSS software package, version 24.0 (SPSS, Chicago). Univariate logistic regression models were used to test the predictive value of all coefficients for the following outcome parameters: (1) the inability conceive again; (2) experience of any further miscarriages, since, empirically, for some women this would be the mostly feared outcome; (3) any live birth > 23 + 0 gestational weeks, since this is the presumably most relevant outcome. Significant parameters were entered in a multivariate logistic regression model. Odds ratios (OR) and their 95% confidence intervals (95% CI) are given. *p* values < 0.05 were considered significant.

## Results

**Fig. 1:**
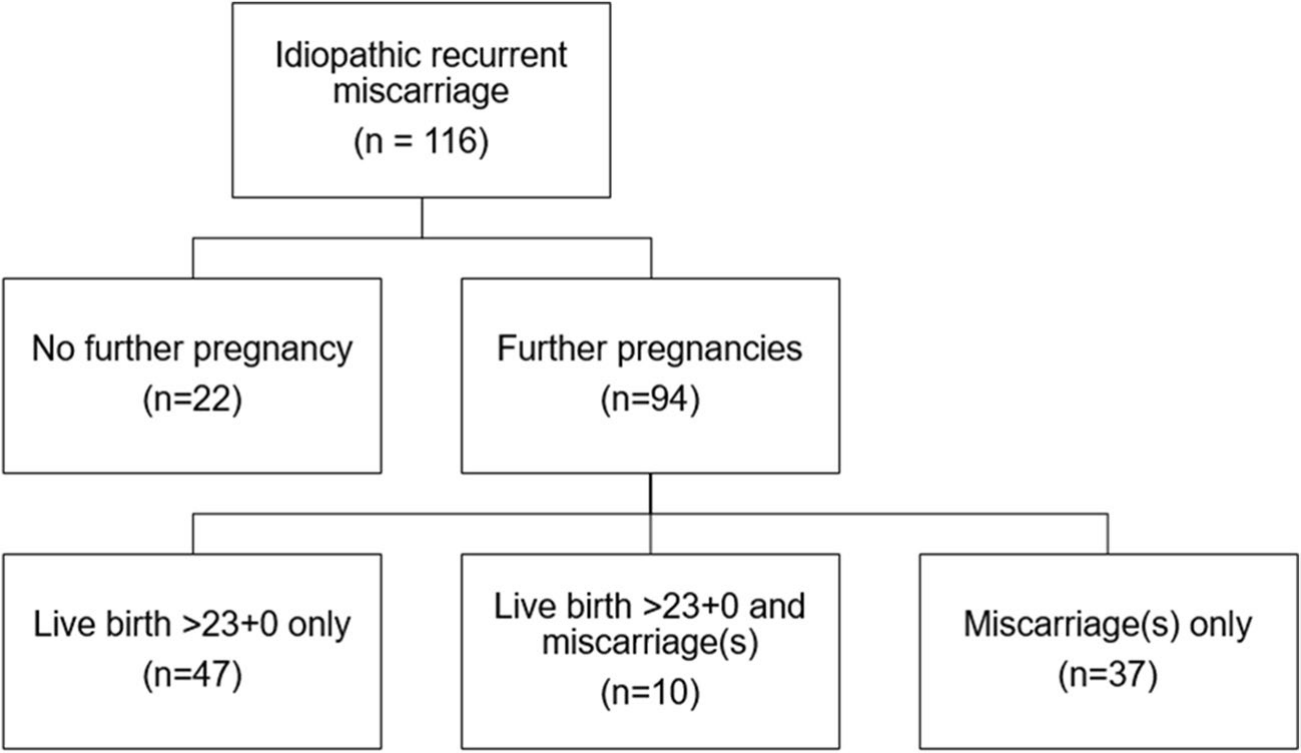
Outcome flowchart

Details on basic patient characteristics are presented in Table 1. A flowchart on further outcomes is presented in Fig. 1. In a first step, women with one or more further pregnancies were compared to those without (Table 2). Higher chronological age and lower AMH levels were associated with a risk of not conceiving again. However, in the multivariate analysis, only age remained statistically significant (OR 1.106, 95% CI 1.005–1.218; *p *= 0.037).

**Table 1:** Basic patient characteristics and results of hormonal testing

Age at diagnostic evaluation (years)^a^	34.1 (28.6; 38.1)
Body mass index (kg/m^2^)^a^	24.0 (21.5; 26.2)
Number of previous early miscarriages^b^	4 (3; 4)
Women with secondary RM^b^	41 (35.3)
LH (IU/L)^a^	5.2 (3.5; 8.2)
FSH (IU/L)^a^	5.5 (3.4; 6.9)
Estradiol (pg/mL)^a^	92 (50; 149)
AMH (ng/mL)^a^	1.6 (0.7; 3.2)
Duration of follow-up (months)^a^	42.3 (22.8; 63.3)
Women with at least one further pregnancy^b^	94 (81.0)
Women with at least one further miscarriage^b^	47 (40.5)
Women with at least one further live birth > 23 + 0^b^	57 (49.1)

Data are presented as ^a^median (interquartile range) for numerical parameters or as ^b^numbers (frequencies) for categorical parameters

**Table 2:** Univariate followed by multivariate analysis for the prediction of the inability to conceive again in women with IRM

	No further pregnancy (*n* = 22)	One or more further pregnancies (*n* = 94)	Univariate analysis		Multivariate analysis	
			Adjusted OR (95% CI)	*p*	Adjusted OR (95% CI)	*p*
Age (years)^a^	38.1 (33.3–41.2)	33.1 (28.2–37.5)	1.141 (1.039; 1.254)	0.006	1.106 (1.005; 1.218)	0.037
Body mass index (kg/m^2^)^a^	24.1 (22.1–26.3)	23.9 (21.5–26.2)	0.994 (0.891; 1.109	0.918	–	–
Number of previous miscarriages^b^	3 (3–4)	3 (3–3)	0.904 (0.698; 1.171)	0.443	–	–
Women with secondary RM^b^	5 (22.7)	36 (38.3)	0.474 (0.161; 1.395)	0.175	–	–
LH (mIU/mL)^a^	4.8 (3.3–7.7)	5.2 (3.5–8.3)	0.981 (0.876; 1.099)	0.743	–	–
FSH (mIU/mL)^a^	5.2 (2.8–8.7)	5.6 (3. 5–6.7)	1.057 (0.914; 1.221)	0.458	–	–
Estradiol (pg/mL)^a^	117 (52–189)	88 (49–133)	1.002 (0.998; 1.006)	0.334	–	–
AMH (ng/mL)^a^	0.6 (1.1–1.6)	1.8 (0.9–3.5)	0.594 (0.392; 0.900)	0.014	0.676 (0.456; 1.003)	0.052
Use of the combined treatment regimen^b,c^	18 (81.8)	74 (78.7)	0.822 (0.209; 3.229)	0.747		
Duration of follow-up (months)^a^	54.3 (22.1–82.6)	36.6 (23.3–62.4)	1.008 (0.996; 1.020)	0.197	–	–

Data are presented as ^a^median (interquartile range) for numerical parameters or as ^a^numbers (frequencies) for categorical parameters; ^c^versus luteal support only

*SD* standard deviation, *RM* recurrent miscarriage

For the next analysis, only those 94 women who achieved one or more further pregnancies were included (Table 3). In this group, only a higher number of previous miscarriages were associated with an increased risk for further miscarriages (OR 3.568, 95% CI 1.457–8.738; *p *= 0.005).

**Table 3:** Univariate followed by multivariate analysis for the prediction of the occurrence of further miscarriages in women with IRM

	Women with further miscarriage(s) (*n* = 47)	Women with a live birth only (*n* = 47)	Univariate analysis		Multivariate analysis	
			Adjusted OR (95% CI)	*p*	Adjusted OR (95% CI)	*p*
Age (years)^a^	33.9 (28.2–37.6)	32.1 (27.5–37.2)	1.049 (0.982; 1.121)	0.157	–	–
Body mass index (kg/m^2^)^a^	24.6 (21.6–26.1)	23.4 (21.4–26.2)	1.001 (0.915; 1.095)	0.978	–	–
Number of previous miscarriages^b^	3 (3–4)	3 (3–3)	3.568 (1.457; 8.738)	0.005	3.568 (1.457; 8.738)	0.005
Women with secondary RM^b^	20 (42.6%)	16 (34%)	1.435 (0.623; 3.307)	0.397	–	–
LH (mIU/mL)^a^	5.0 (3.2–7.9)	5.4 (3.9–8.7)	0.937 (0.846; 1.038)	0.21	–	–
FSH (mIU/mL)^a^	5.5 (3.5–6.7)	5.9 (3.1–7.2)	0.990 (0.853; 1.149)	0.894	–	–
Estradiol (pg/mL)^a^	85 (49–129)	88 (50–159)	0.998 (0.994; 1.002)	0.206	–	–
AMH (ng/mL)^a^	1.8 (0.8–3.1)	1.7 (0.9–3.7)	0.893 (0.743; 1.074)	0.230	–	–
Use of the combined treatment regimen^b,c^	32 (68.1)	38 (80.9)	0.505 (0.195; 1.307)	0.159	–	–
Duration of follow-up (months)^a^	32.5 (19.2–61.4)	42.3 (26.6–62.7)	0.991 (0.978; 1.005)	0.195	–	–

Data are presented as ^a^median (interquartile range) for numerical parameters or as ^b^numbers (frequencies) for categorical parameters; ^c^versus luteal support only

*SD* standard deviation, *RM* recurrent miscarriage

The third analysis included all 116 women and dealt with the chance to achieve a live birth > 23 + 0 gestational weeks (Table 4). In the univariate model, lower age, lower number of previous miscarriages, and higher AMH levels were associated with an increased chance of a future live birth. In the multivariate analysis, only age (OR 0.920, 95% CI 0.859–0.986; *p *= 0.019) and the number of previous miscarriages (OR 0.403, 95% CI 0.193–0.841; *p *= 0.016), but not AMH (OR 1.191, 95% CI 0.972–1.461; *p *= 0.091), remained statistically significant.

**Table 4:** Univariate followed by multivariate analysis for the prediction of live birth > 23 + 0 gestational weeks

	Women with a further live birth (*n* = 57)	Women without a further live birth (*n* = 59)	Univariate analysis		Multivariate analysis	
			Adjusted OR (95% CI)	*p*	Adjusted OR (95% CI)	*p*
Age (years)^a^	32.1 (27.5–37.1)	36.3 (31.7–39.8)	0.908 (0.851; 0.968)	0.004	0.920 (0.859; 0.986)	0.019
Body mass index (kg/m^2^)^a^	23.7 (21.5–26.3)	24.1 (21.6–26.1)	1.000 (0.919; 1.088)	0.997	–	–
Number of previous miscarriages^b^	3 (3–3)	3 (3–4)	0.424 (0.213; 0.846)	0.015	0.403 (0.193; 0.841)	0.016
Women with secondary RM^b^	19 (33.3)	22 (37.3)	0.841 (0.392; 1.803)	0.656	–	–
LH (mIU/mL)^a^	5.4 (3.9–8.7)	4.9 (3.2–7.8)	1.037 (0.949; 1.132)	0.421	–	–
FSH (mIU/mL)^a^	5.9 (3.3–8.7)	5.2 (3.4–7.0)	1.011 (0.895; 1.142)	0.863	–	–
Estradiol (pg/mL)^a^	85 (50–151)	100 (49–151)	1.001 (0.997; 1.005)	0.481	–	–
AMH (ng/mL)^a^	1.9 (0.9–3.6)	1.3 (0.4–2.5)	1.245 (1.029; 1.505)	0.024	1.191 (0.972; 1.461)	0.091
Use of the combined treatment regimen^b,c^	48 (84.2)	44 (74.6)	1.818 (0.724; 4.569)	0.204	–	–
Duration of follow-up (months)^a^	43.3 (27.3–63.1)	33.9 (19.2–69.2)	1.005 (0.993; 1.017)	0.326	–	–

Data are presented as ^a^median (interquartile range) for numerical parameters or as ^b^numbers (frequencies) for categorical parameters; ^c^versus luteal support only

*SD* standard deviation, *RM* recurrent miscarriage

## Comment

Although women with RM, and especially those with IRM, show lower AMH levels according to recent studies [1, 2], AMH seems to play only a minor role in the prediction of further outcomes in women with IRM. Obviously, the explanatory power of the woman’s age was higher than that of AMH. Notably, AMH levels correlate with the size of the follicle pool rather than with the quality of the oocytes. This is also reflected by the fact that AMH does not predict live birth after IVF whereas age does [15]. The present retrospective study suggests that the affected woman’s age and the number of previous miscarriages have the highest impact. These findings are generally in good accordance with those of previous studies [16–18], although a recently published large study was unable to confirm the effect of the number of previous miscarriages [19].

Given the detrimental impact of RM on psychological well-being, some women might be more interested in avoiding further miscarriages rather than in their chances for a live birth. For these patients, only the number of previous miscarriages was of predictive value in our dataset (Table 3), allowing only a quite unreliable prediction. The chance for a live birth was 60.6% (57/94). It has already been reported that women with IRM would have a good outcome for subsequent pregnancies [17, 19, 20]. Notably, it has been demonstrated that there was no difference in prognosis between women with RM due to a known cause and those with IRM [19, 21]. As reported recently, this might be due either to the fact that pathologies in RM due to a known cause are treated very effectively, or that the definition of IRM is insufficient [19]. However, despite the fact that chronological age and markers for ovarian reserve seem to be associated with IRM [1, 2], they were not predictive for further miscarriages in the present dataset. This could be due to the small sample size; however, in view of the odds ratios for age and AMH, the impact of these factors would remain only modest even if statistical significance was to be reached in larger data sets. This suggests that the criteria for IRM might indeed be lacking [19]. Accordingly, other factors must be of greater importance. In this context, we consider it a minor study limitation that we cannot provide details on modifiable risk factors, including alcohol consumption, lifting of heavy weights, and night work [22], which is attributable to the retrospective study design.

However, for many affected couples, the main focus might be on the chances for a future live birth. A lower chronological age and a lower number of previous miscarriages favored this outcome (Table 4). Although it did not quite reach statistical significance, there was at least a trend for an association between AMH and future live birth (*p *= 0.091). It must be mentioned that this analysis also included those women who were unable to conceive again, an outcome that could best be predicted by age and AMH (Table 2). Thus, it seems reasonable that a higher chronological/biological age would affect this outcome. Nonetheless, the effect size was only moderate, which again reflects the considerations mentioned above in the discussion about the prediction of further miscarriages.

Two factors that were not influential for any of the outcome parameters in the present dataset need to be discussed. First, a history of a live birth, i.e., “secondary RM,” did not influence the risk of a further miscarriage, nor the chance for live birth. This is in accordance with the previous results [17]. One possible reason is the fact that only women with IRM were included in the present study. In addition, it could be, again, postulated that many unknown factors exist in women with IRM that ultimately influence outcome. Second, some women chose the combined treatment regimen for IRM which has been recommended in previous studies [11, 23, 24]. However, we are aware of the fact that this combined treatment regimen is not according to the international standards. Notably, according to Table 3, the combined treatment regimen did not influence outcome in our study population. Since it has been included as an independent parameter in the uni- followed by multivariate analyses, we consider its use a minor study limitation.

The latter must be seen as a study limitation, as is the retrospective design. Furthermore, the use of the Beckman AMH ELISA might be seen as an additional minor study limitation, since it has been discussed as inferior due to a worse analytical performance [25]. Moreover, values measured before August 2013 had to be corrected using a special formula. This also underlines the necessity to prove our results in future studies with other tools for AMH measurement. Moreover, one could argue that achievement of a live birth before or after another miscarriage, might imply something different than if a patient would have a live birth only. We are unable to provide analyses on these patient subgroups due to the sample size. However, to the best of our knowledge, this is the first study to evaluate AMH as a possible predictive factor for outcomes in RM. Larger prospective trials are warranted to confirm our findings.

In conclusion, despite AMH’s value in assessing outcome of artificial reproductive techniques, it seems to be of either no or only minor relevance for the prediction of further miscarriages and live birth in women with IRM.
